# Chicken pituitary transcriptomic responses to acute heat stress

**DOI:** 10.1007/s11033-023-08464-8

**Published:** 2023-05-02

**Authors:** Elizabeth M. Pritchett, Angelica Van Goor, Blair K. Schneider, Meaghan Young, Susan J. Lamont, Carl J. Schmidt

**Affiliations:** 1 Animal and Food Science, University of Delaware, Newark, DE USA; 2 Animal Science, Iowa State University, Ames, IA USA; 3 Food Science and Human Nutrition, Iowa State University, Ames, IA USA

**Keywords:** Pituitary, Chicken, Heat stress, Transcriptome, Stress response, Metabolism

## Abstract

**Background:**

Poultry production is vulnerable to increasing temperatures in terms of animal welfare and in economic losses. With the predicted increase in global temperature and the number and severity of heat waves, it is important to understand how chickens raised for food respond to heat stress. This knowledge can be used to determine how to select chickens that are adapted to thermal challenge. As neuroendocrine organs, the hypothalamus and pituitary provide systemic regulation of the heat stress response.

**Methods and Results:**

Here we report a transcriptome analysis of the pituitary response to acute heat stress. Chickens were stressed for 2 h at 35 °C (HS) and transcriptomes compared with birds maintained in thermoneutral temperatures (25 °C).

**Conclusions:**

The observations were evaluated in the context of ontology terms and pathways to describe the pituitary response to heat stress. The pituitaries of heat stressed birds exhibited responses to hyperthermia through altered expression of genes coding for chaperones, cell cycle regulators, cholesterol synthesis, transcription factors, along with the secreted peptide hormones, prolactin, and proopiomelanocortin.

**Supplementary Information:**

The online version contains supplementary material available at 10.1007/s11033-023-08464-8.

## Introduction

Poultry production, particularly broiler (meat-type) production, is an important component of United States Agriculture. In 2021, approximately 9.1 billion broilers were produced valuing 31 billion dollars [1]. Heat stress is an environmental stressor that leads to animal welfare concerns such as increased morbidity and mortality along with decreased feed intake and feed efficiency. In production settings, these responses ultimately lead to significant economic losses. The selection for increased growth in broiler chickens has led to the decreased efficiency of other systems (i.e. cardiovascular and respiratory) and increased susceptibility to high ambient temperatures [2, 3]. The Intergovernmental Panel on Climate Change predicted an increase in the global average temperatures of 1.8 °C to 4.0 °C by the year 2100 and an increase in the number and intensity of heat waves [4]. The effects of heat stress in poultry production may become more apparent as the global mean temperature continues to increase [5].

Several processes are affected when birds are exposed to increased temperatures including panting to release heat through the respiratory tract along with decreased feed consumption and movement. In addition to these behavioral changes, multiple physiological processes are affected including blood flow, metabolism, stress response, and immune function. At the cellular level, heat stress impacts the cell cycle, DNA repair mechanisms, transcription, translation, post-translational modifications, oxidative metabolism, membrane structure and function, and the unfolding or improper folding of proteins [6, 7]. Heat shock proteins (HSP) are highly conserved molecular chaperones that assist in the cellular response to heat stress in several ways including intracellular transport, protein folding, prevention of protein denaturation, prevention of protein aggregation, and facilitation of protein renaturation. Many HSP encoding genes respond to heat stress by increased transcription and translation during times of heat stress [8, 9].

The neuroendocrine system encompasses the hypothalamus and pituitary, which work together to regulate physiological processes such as responses to stress, growth, metabolism, and reproduction. Although selection of the broiler for increased growth and feed efficiency has been extremely successful, the processes of the neuroendocrine system are still not fully understood [10]. In prior work, we have characterized the pituitary transcriptome of broiler chicks during post-hatch development [11]. In this work we identify differentially expressed pituitary genes by comparing transcriptomes between chickens exposed to thermoneutral conditions or 2 h of exposure to heat stress. The aim of this study is to identify differentially expressed genes in the pituitary to better understand the genetic and pathway responses to increased ambient temperatures in broiler chickens. This manuscript was previously submitted as a pre-print to Research Square (https://doi.org/10.21203/rs.3.rs-2574121/v1).

## Methods

### Ethics statement

This study was carried out in strict accordance with the recommendations in the Guide for Care and Use of Laboratory Animals of the National Institutes of Health. The protocol was approved by the Committee on Ethics and Animal Experiments of the University of Delaware (permit number: 2703–12–10).

### Animal housing and tissue collection

Male Ross 708 broiler chickens (*Gallus gallus)* were obtained on day of hatch from Mountaire Hatchery (Millsboro, DE) and placed in houses on the University of Delaware farm (Newark, DE). Birds were provided with standard broiler feed (corn-soy) that met all NRC requirements [12]; food and water were supplied ad libitum. At day 26 post-hatch (D26), six chickens were exposed to acute heat stress for 2 h by moving these birds from a thermoneutral house at 25 °C to one maintained at 35 °C. After 2 h, the six heat stressed birds and an additional six birds from the thermoneutral house were euthanized by cervical dislocation, the pituitary glands collected and immediately flash frozen with liquid nitrogen and stored at − 80 °C until further processing. To examine the impact of moving the birds between houses in the absence of heat stress, at D26 five chickens were moved from one thermal neutral house to a second thermal neutral house that contained the same number of birds as the heat stress house. After 2 h, pituitaries were obtained from the moved birds along with five birds that were not moved from the original house (unmoved birds) and transcriptome analysis performed. Our facilities do not have humidity control. The average humidity on the Delmarva peninsula in the spring is 50%.

### RNA isolation, cDNA synthesis, and RNA-seq library preparation

Total RNA was extracted from whole pituitary glands (6 mg) using the Qiagen RNeasy Mini Kit (Germantown, MD). Total RNA quantity was measured using a Qubit Fluorometer and quality was assessed via fragment analysis. A total of 6 pituitary glands from heat stress birds and 6 from thermoneutral birds were obtained for RNA sequencing (RNA-seq) library preparation using the Illumina Stranded RNAseq kit (San Diego, CA). Transcriptome libraries were also prepared from five birds that remained in the thermoneutral house (unmoved) and five that were moved to a second thermoneutral house. During library preparation, one heat stressed sample yielded a low-quality library and was not sent for sequencing (Bioanalyzer fragment size < 100 bp). Five heat-stressed and 6 control samples were sent for sequencing at the Delaware Biotechnology Institute Sequencing and Genotyping center using the Illumina HiSeq 2500 sequencer. All libraries were sequenced to a depth of ~ 20 to 30 million reads. The reads were aligned to the *Gallus gallus* ver6 genome sequence, identified and counted using the Tuxedo software package to determine fragments per kilobase of gene per million mapped reads (FPKM) values for further analysis [13, 14].

### Differentially expressed genes (DEG) analysis

Raw FPKM values for all 24,356 transcripts from all experiments can be found in Table S1. Differential gene expression allows for the comparison of expressed genes between two conditions (Heat Stress versus Thermoneutral). Based on Schurch et.al. [15] five replicates and our depth of sequencing provided sufficient power to detect > 75% of differentially enriched genes at a p-value of 0.05 or lower. Using the *Gallus gallus* reference genome gal6, DEG were identified following the protocol outlined by Davis et al., 2015 [16]. Mean FPKM ratios were compared between heat stress and thermoneutral birds for all genes. A log_2_ transformation was used to normalize data and a t-test was applied. Genes whose ratios were greater than 2 standard deviations from the mean and had an FDR < 0.05 were considered differentially expressed between heat stress and thermoneutral conditions. The resulting DEG with FPKM values greater than 1 in at least one condition (heat stress and/or thermoneutral) were uploaded to GoNet for gene ontology analysis [17].

### Quantitative reverse transcription polymerase chain reaction (qRT-PCR) verification

Three biological replicates for each condition were used for cDNA synthesis using the SuperScript First-Strand Synthesis System for RT-PCR (Invitrogen). cDNA concentration was determined using the Qubit Fluorometer and diluted to 30 ng/ul for PCR. qRT-PCR was performed using Fast SYBR green master mix (Applied Biosystems) on the Applied Biosystems 7500 Fast Real Time PCR System for the following genes: *Growth Hormone* (GH), *Proopiomelanocortin* (POMC), and *Heat Shock Protein 90 kDa Alpha Family Class A Member 1* (HSP90AA1). The remaining genes, *Heat Shock Protein Family A (HSP70) Member 2* (HSPA2), *Heat Shock Protein Family H (Hsp110) Member 1* (HSPH1), and *BCL2 Associated Athanogene 3* (BAG3) were verified utilizing the Fluidigm Biomark HD microfluidic device as outlined in Van Goor et al., 2016 [18]. Each gene and primer pair (Table 1) were performed in triplicate and analysis was completed using the delta-delta Ct method [19].

**Table 1:** Primer sequences used for qRT-PCR transcriptome validation of *chicken* differential expression between heat stress and thermoneutral conditions

Gene	Forward primer	Reverse primer	qRT-PCR method
GH	5′ GCTTCAAGAAGGATCTGCACAA 3′	5′ GCGCCGGCACTTCATC 3′	Fast SYBR
POMC	5′ GCTACGGCGGCTTCATGA 3′	5′ CGATGGCGTTTTTGAACAGA 3′	Fast SYBR
HSP90AA1	5′ GCAGCAGCTGAAGGAATTTGA 3′	5′ GGAAGCTCTAAGCCCTCTTTTGT 3′	Fast SYBR
RPL4	5′ TCGCCCTGATGTGGTGAA 3′	5′ GCATAGGGCTGCCTGTTGTT 3′	Fast SYBR
BAG3	5′ ACCACAACAGCCGAACCA 3′	5′ GATGGGCCATTTGCTGATGAC 3′	Fluidigm Biomark
HSPA2	5′ CCACCATTCCCACCAAACAA 3′	5′ ATACACCTGGACGAGGACAC 3′	Fluidigm Biomark
HSPH1	5′ GTAGTTTCGTTCGGCTCCAA 3′	5′ CTGTGTTGTGGGCATGAGTAA 3′	Fluidigm Biomark
RPL4	5′ TTCTGCCTTGGCAGCATCA 3′	5′ AGGAAGTTCTGGGATCTCCTCA 3′	Fluidigm Biomark

*GH* growth hormone, *POMC* pro-opiomelanocortin, *HSP90AA1* heat shock protein 90 kDa alpha, class A member 1, *RPL4* ribosomal protein L4, *BAG3* BCL2 associated athanogene 3, *HSP70* heat shock protein family A, *HSPA2* Member 2, *HSP110* heat shock protein family H, *HSPH1* Member 1. RPL4 is reference gene for both methods

## Results

**Fig. 1:**
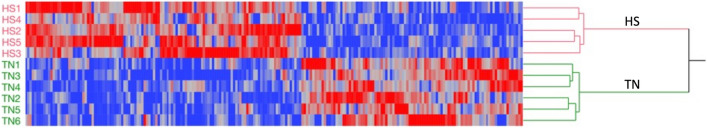
Hierarchical clustering of 95 significant differentially expressed genes between 2-h heat stress and thermoneutral conditions in the chicken pituitary gland. Dendrograms are in arbitrary distance scale. Heat stress (HS) and thermoneutral (TN) samples segregate independently. Red indicates transcripts elevated and blue those reduced under the indicated conditions (HS or TN)

Expression levels of 24,356 genes were analyzed to identify differentially expressed genes (DEG). A total of 95 genes were differentially expressed between conditions with 36 down regulated and 59 up regulated in response to heat stress (FDR < 0.05) (Table S2). Hierarchical clustering segregated the DEG by condition (Fig. 1). qRT-PCR gave the same direction of change for heat-responsive genes (BAG3, HSPA2, HSPH1, HSP90AA1, and POMC) as was seen in the transcriptome data.

### Gene ontology analysis

**Fig. 2:**
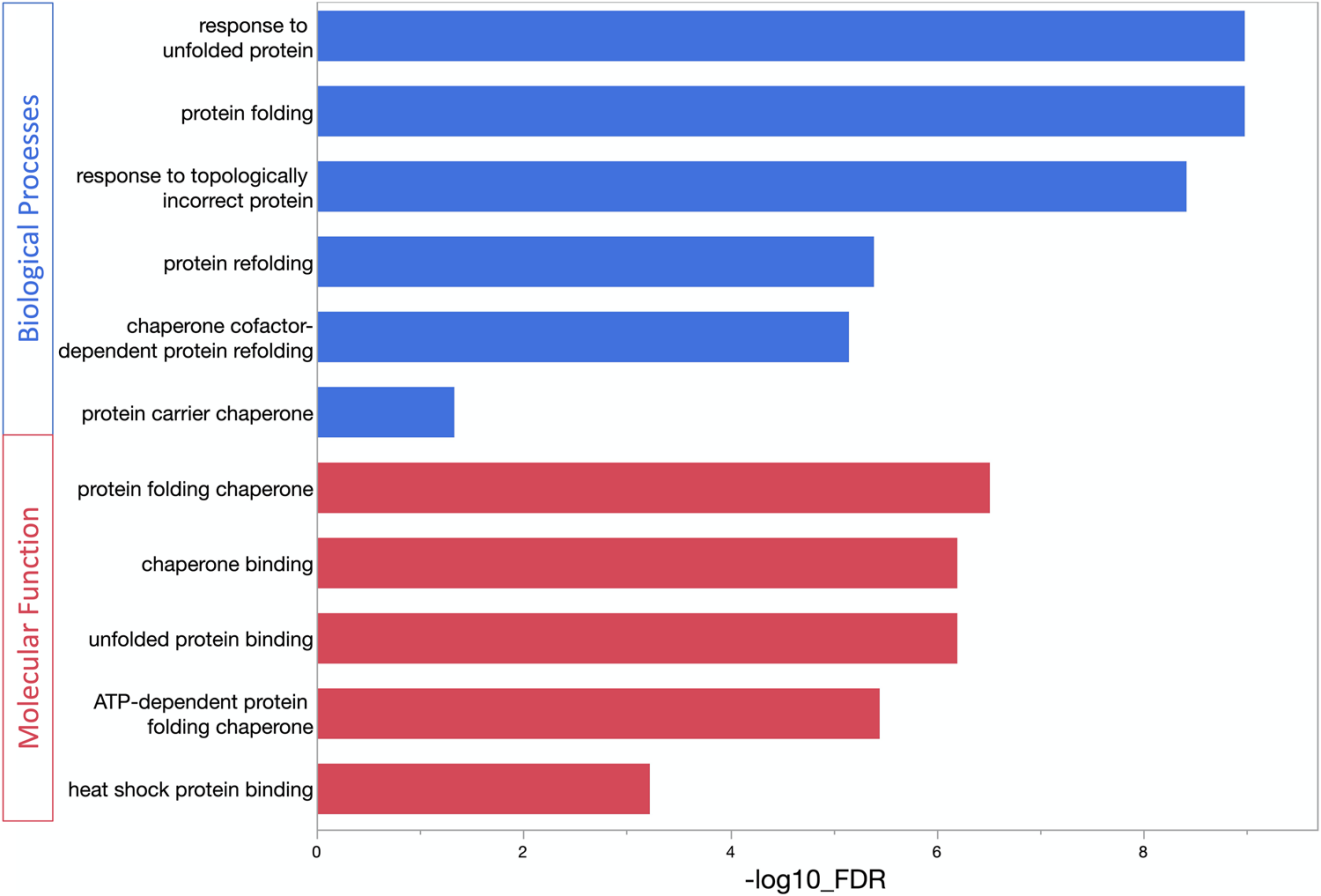
Gene ontology terms enriched by the heat stress responsive differentially expressed genes. Blue histograms refer to the Biological Process Ontology while the red correspond to the Molecular Function Ontology. The Y axis refers to specific GO categories and the X axis the -log10 of the false discovery rate

The Gene Ontology (GO) was used to identify enriched terms associated with the differentially expressed genes (Fig. 2). Consistent with the treatment, the enriched biological processes and molecular functions highlighted protein folding and chaperone function. These terms are typical of cellular or tissue responses to heat stress. Other GO terms did not reach significance, as might be expected given the small number of genes exhibiting differential expression under these conditions.

### Stress response genes

The Gene Ontology term having the highest network degree was “Response to Stress” which included 4 down regulated and 22 up regulated genes (Table 2). Of these, 14 upregulated genes function as part of the chaperone system that is responsible for proper protein folding and play an important role in maintaining cell viability during heat stress. Transcripts encoding seven heat shock proteins (HSP) were enriched following 2 h of acute heat stress. HSP90AA1, HSPA2, HSPA4L, HSPA5, AND HSPA8 are chaperones. HSP90AA1 belonging to the HSP90 class and the HSPA group belong to HSP70 class. HSPH1 is a co-chaperone that acts as a nucleotide exchange factor controlling the activity of HSP70 proteins. Members of the DNAJ family also function as heat shock proteins. DNAJA4 also modulates cholesterol and reactive oxygen synthesis [20]. DNAJB1 promotes ubiquitination and proteasomal degradation of misfolded proteins and is responsible for suppressing p53 mediated apoptosis [21]. DNAJB5 functions as a co-chaperone for the HSP90 chaperones [22]. *Jumonji domain containing 6* (JMJD6) prevents apoptosis by catalyzing the hydroxylation of TP53 and promotes TP53 association with its negative regulator MDM4, thereby repressing TP53’s transcriptional activity [23].The calreticulin (CALR) transcript encodes another chaperone that is elevated in the heat stressed pituitary. Among its many roles, CALR serves as a chaperone to guide the correct folding of glycoproteins within the endoplasmic reticulum [24, 25].

**Table 2:** Gene Ontology terms associated with differentially expressed genes

Term	Genes
Response to Stress*	BAG3, CALR, DNAJA4, DNAJB1, DNAJB5, HERPUD1, HSP90AA1, HSPA2, HSPA4L, HSPA5, HSPA8, HSPB8, HSPH1, POFUT2, PLK3
Apoptosis	BAG3, CALR, HERPUD1, HSP90AA1, HSPA5, ID1, JMJD6, PHLDA2, PLK3, PPP1R10, SENP1, ZBTB16, ZMYND11
Autophagy	BAG3, DNAJB1, HSP90AA1, HSPA8, PLK3, TP53INP
Transcription	BAG3, CALR, DNAJB1, GSX1, HR, HSP90AA1, HSPA5, HSPA8, ID1, JMJD6, KAT2A, H2AFY2, PLK3, POMC, PRL, SENP1, SOX17, TBX18, TBX22, TP53INP2, ZBTB16, ZMYND11
Cell Cycle^#^	CALR, GINS2, HSP90AA1, HSPA2, HSPA8, KAT2A, PLK3, STAG1, ZMYND11

*Including “stress”, “folding”, “unfolded”, and “chaperone”

#Including “cell cycle” and “centrosome”

### Protein modification

Protein modifications such as ubiquitination, sumoylation, methylation or phosphorylation typically act as toggle switches where activities change as a function of the modification state of the substrate. *Homocysteine inducible ER protein with ubiquitin like domain 1* (HERPUD1) is an endoplasmic reticulum protein involved in the endoplasmic reticulum-associated degradation complex (ERAD) and is essential for neuronal survival [26]. HERPUD1 protects the cell from apoptosis by delivering ubiquitinated substrates to the proteasomes [27]. HERPUD1 also stabilizes ER and mitochondrial calcium levels as a mechanism of avoiding apoptosis [28].

SUMO specific peptidase 1 (SENP1) catalyzes the addition of the SUMO protein to lysine residues of target proteins. SUMO and ubiquitin proteins are structurally similar and exhibit similar functions. Sumoylation involves the initial addition of SUMO to target proteins followed by a series of modifications to the SUMO moiety. Multiple targets for sumoylation have been identified, including proteins that control DNA repair and ERAD along with ones modulating transcription [29]. For example, HSF1, the main transcription regulator activated by heat stress, is modified by sumoylation [30].

Lysine acetyltransferase 2A (KAT2A) binds acetyl CoA and functions as a histone acetyltransferase. Acetylated histones typically mark regions of open chromatin that are actively transcribed. In humans, Chip-seq data (retrieved from online resource published in [31] on 09/28/2022) has shown KAT2A interacting with multiple genes responsive to heat stress including HSP90AA1, HSPA4L, HSPA5, HSPA8, HSPH1, BAG3 AND CHORDC1, all of which were induced in the pituitary during the 2-h heat stress trial. Jumonji domain containing 6 (JMJD6) is an arginine demethylase targeting HSPA proteins [32] and also functions as a lysine hydroxylase [33]. The latter activity has been implicated in the hydroxylation of proteins that form membrane-less organelles such as stress granules, transcriptional condensates, and spliceosomes. Lysine demethylase and nuclear receptor corepressor (HR) is a histone demethylase that represses the activity of several receptor genes including the thyroid [34] and vitamin D receptors [35]. Polo Like Kinase 3 (PLK3) functions as a chaperone for multiple nuclear factors including TP53. For example, PLK3 phosphorylates TP53 activating the transcription mediated DNA repair pathway [36]. In addition, PLK3 phosphorylation of HSP90 promotes degradation of this substrate [37].

Zinc Finger DHHC-Type Palmitoyltransferase 2 (ZDHHC2) regulates the sub cellular localization of proteins by addition of lipid moieties. Prolyl 4-hydroxylase subunit alpha 2 (P4HA2) plays an important role in the maturation of collagen. By hydroxylating proline residues of procollagen, the enzyme promotes the proper folding of collagen. Methyltransferase-Like 27 (METTL27) belong to a family of methyltransferase-like proteins and family members modify a variety of substrates including proteins, DNA and RNA [38]. Currently, the biological role of METTL27 is unknown.

### Apoptosis and autophagy

Apoptosis, an organized pathway for cell death, is regulated by several differentially regulated genes. The apoptosis inhibitors, HERPUD, DNAJB5, and JMJD6, have already been described. Additional apoptosis inhibitors include: BAG3 [39], HSP90AA1 [40], HSPA5 [41], ID1 [42] PPP1R10 [43] and SENP1 [44], while ZBTB16 [38] activates apoptosis. Autophagy is a lysosomal based degradation pathway with specific targets that are recycled for cellular maintenance. This pathway is responsive to heat stress and plays a role in maintaining thermotolerance [45]. HSPB8 and HSPA8 are co-chaperones that promote autophagy in complex with BCL2 associated athanogene 3 (BAG3) chaperone [46]. BAG3 is a co-chaperone involved in protein refolding and showed 11-fold difference between HS and TN conditions. BAG3 is known to decrease apoptosis and increase autophagy during heat stress [47]. Three additional transcripts encoding proteins activating autophagy were up regulated by heat stress: DNAJB1, which serves as a chaperone in the formation of the autophagosome [48]. VIPAS39 controls lysosomal sorting [43] and LGALS8 is a membrane damage sensor [49–52]. Pleckstrin homology like domain family A member 2 (PHLDA2) negatively regulates autophagy, and the transcript encoding this protein was enriched by heat stress [53]. Two transcripts affecting autophagy were downregulated: EMC6, a component of the autophagosome membrane [54] and TP53INP2 a scaffolding protein that functions in autophagy membrane formation [55].

### Cholesterol and lipid metabolism

Sterol-C5-Desaturase (SC5D) transcripts are elevated in response to heat stress. SC5D catalyzes the conversion of Lathosterol to 7-Dehydrocholesterol, which is the penultimate metabolite in the Kandutsch-Russell pathway for cholesterol synthesis. While not reaching our FDR < 0.05, cutoff, the transcript levels encoding three other enzymes critical to cholesterol synthesis were elevated under heat stress including: 3-Hydroxy-3-Methylglutaryl-CoA Reductase (HMGCR; HS mean FPKM = 191.4, TN FPKM mean = 122.9); 24-Dehydrocholesterol Reductase (DHCR24; HS mean FPKM = 54.1, TN FPKM mean = 39.7) and 7-Dehydrocholesterol Reductase (DHCR7; HS mean FPKM = 136.2, TN FPKM mean = 97.7). The increase in the transcripts encoding these genes is consistent with reports of cholesterol levels increasing during heat stress [56–58]. As mentioned above, DNAJA4 also affects cholesterol metabolism by increasing HMGCR protein levels [20].

Three transcripts implicated in lipid metabolism were also up-regulated by heat stress: 1-acylglycerol-3-phosphate O-acyltransferase 1 (AGPAT1), probable very-long-chain enoyl-CoA reductase art-1 (TECR), and transmembrane protein 159 (TMEM159). AGPAT1 catalyzes the conversion of lysophosphatidic acid to phosphatidic acid. These substrates function as precursors to other lipids and can act as second messengers controlling membrane dynamics including fusion and fission. The TECR gene likely encodes the enzyme responsible for addition of two carbon units to long or very-long fatty acids. TMEM159 initiates the formation of lipid droplets that store triacylglycerols and cholesterol esters [59]. Lipid droplets provide membrane components during cell growth and proliferation and can deliver lipids to mitochondria, peroxisomes, or lysosomes for metabolism.

### Cell cycle

**Fig. 3:**
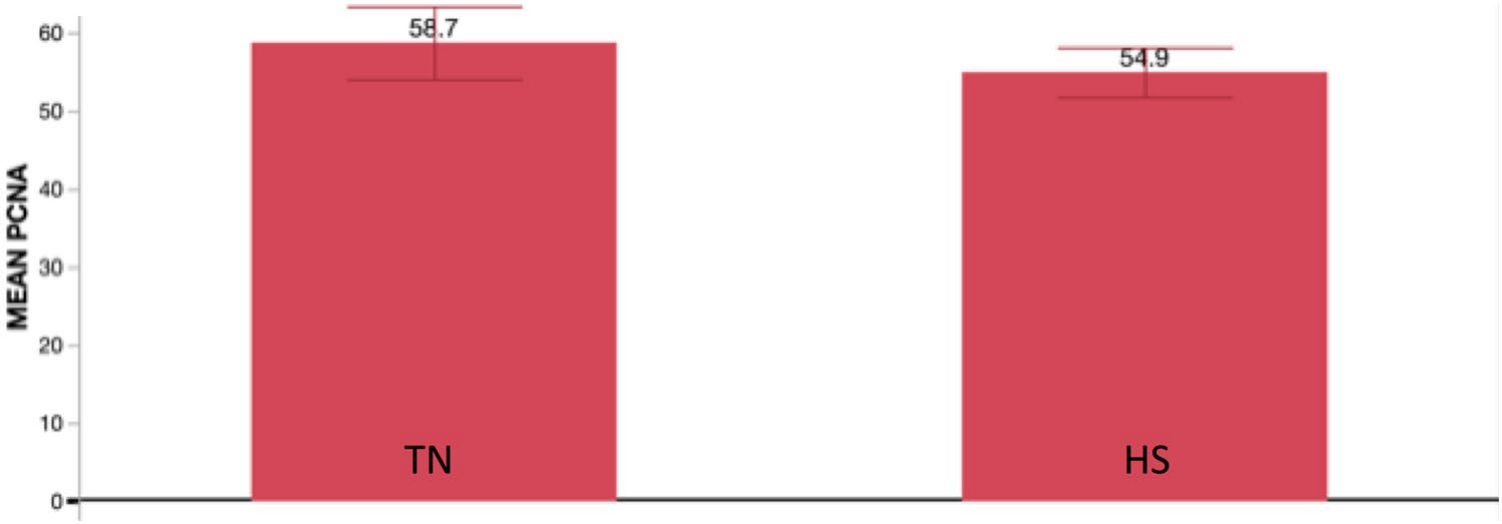
Mean levels of proliferating cell nuclear antigen (PCNA) transcript levels (FPKM) in pituitary from chickens raised under thermoneutral (TN) or heat stressed (HS) conditions

In the pituitary a small number of genes GO annotated as impacting the cell cycle or DNA repair were differentially regulated in response to heat stress. DEGs affecting the cell cycle include: CALR, HSP90AA1, HSPA2, HSPA8, PLK3, STAG1, GINS complex subunit 2 (GINS2), lysine acetyltransferase 2A (KAT2A), and zinc finger MYND-type containing 11 (ZMYND11). Those affecting DNA repair include HSPA5, PLK3, STAG1, and serine/threonine-protein phosphatase 1 regulatory subunit 10-like (PP1R10). The pituitary was sampled on day 26 post- hatch, when the birds are still actively growing. To verify that there were proliferating cells within the pituitary the transcriptome analysis was examined for the level of proliferating cell nuclear antigen (PCNA). While there was no significant difference between the heat stress and thermoneutral conditions, PCNA expression was readily detected, indicating that proliferating cells were present in the pituitaries (Fig. 3).

### Peptide hormones

Consistent with another report, Prolactin (PRL) was enriched 70-fold in the heat stress condition [60]. It is unclear what impact elevated PRL levels would have under heat stress. Along with PRL, there was a fivefold enrichment of Proopiomelanocortin (POMC) expression in heat stress birds. POMC is a precursor peptide that is cleaved to yield adrenocorticotropic hormone (ACTH) which increases corticosterone, the main glucocorticoid in birds. This has many downstream effects including an increase of anti-inflammatory proteins, gluconeogenesis, lipolysis, and appetite suppression [61].

### Transcription regulators

Seven transcripts encode proteins that directly modulate transcription. Five were down-regulated (ID1, HR, TBX22, TBX18, SOX17) while three were up-regulated (ZMYND11, ZBTB16 (described above), H2AFY2). Inhibitor of DNA Binding (ID1) lacks a DNA binding domain but interacts with basic HLH DNA binding proteins and inhibits their activation functions [62]. HR interacts with TP53 and promotes cell cycle arrest and apoptosis. Both T-Box Transcription factor 22 (TBX22) and TBX18 bind DNA, typically inhibit transcription, and are best characterized for their roles in early development [63]. SRY-box transcription factor 17 (SOX17) is also best characterized for its role in early development and can function as a transcription activator or repressor depending on the identity of its dimeric binding partner [64]. Zinc finger MYND-type containing 11 (ZMYND11) is a multidomain protein that can modulate RNA polymerase activity [65] and regulates splicing [66]. H2A Histone Family Member Y2 (H2AFY2) is a variant histone typically associated with inactive chromatin. The biological reasons underlying the differential response of these transcription regulators to heat stress are unclear.

An additional 13 differentially expressed genes affect transcription. Two were down regulated (GSX1 and TP53INP2) while 11 were up-regulated (CALR, PLK3, SENP1, JMJD6, HSP90AA1, HSPA8, KAT2A, HSPA5, DNAJB1, BAG3, and STAG1). Except for STAG1, the encoded proteins impact transcription indirectly, either by guiding proper folding or by modifying transcription factors. STAG1 is a functional component of the Cohesin complex which is important in the cell cycle and serves to generate chromatin topologically associated domains that regulate transcription [67].

### lncRNA expression

A total of ten lncRNAs were differentially expressed with three being down-regulated and seven up-regulated in the heat stress samples. Two heat stress enriched lncRNAs had high positive correlation (r > 0.9) with multiple protein coding genes. lncRNA_ENSGALG00000037064 showed high correlation with the expression levels of HSP90AA1, KAT2A, DNAJB5, DNAJB1, BAG3, HSPA2, CHORDC1, and DNAJA4. lncRNA_ENSGALG00000050713 was highly correlated with RAB18L, PPP1R10, HSPA5, HSPA4L, DNAJA1, and HSPA8.

### Stress arising from moving birds

**Fig. 4:**
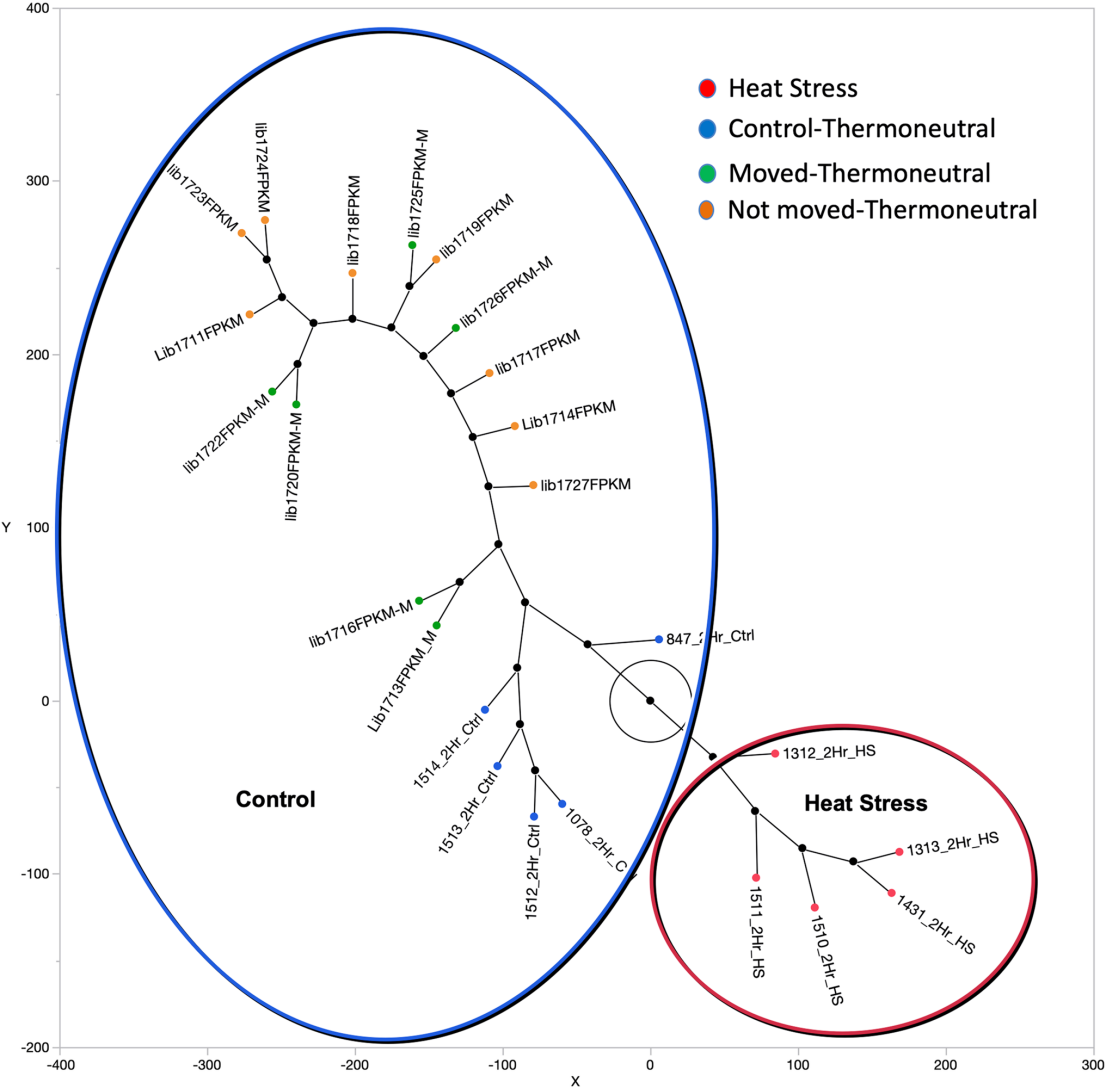
Hierarchical Clustering showing two distinct clusters, one containing the birds subjected to acute heat stress (red oval) with the second containing all birds maintained at thermoneutral conditions (blue oval). The birds in the second group included those that remained in the thermal neutral house and those that were moved between two thermoneutral houses. X and Y coordinates are distance units

Any changes in gene expression detected between the moved and unmoved birds may represent transcriptome changes due to stress of moving and being introduced to a new flock. In a separate experiment, transcriptome analysis comparing birds moved between thermoneutral houses and the unmoved birds identified 50 differential expressed transcripts (Table S3). Hierarchical clustering showed that the moved and unmoved birds are more like the thermoneutral birds from the original heat stress experiment (Fig. 4). All birds maintained at thermoneutral conditions (moved and unmoved) were distinct from the birds exposed to heat stress. Enrichment analysis failed to identify any enriched Gene Ontology Terms, KEGG or Reactome Pathways, suggesting that moving these birds between houses had little effect on the genes identified as responsive to heat stress.

## Discussion

The pituitary is central to monitoring internal physiology and physiology affected by environmental challenge. As a consequence of the monitoring function this organ coordinates its own response to maximize survival of the tissue. The pituitary also releases peptide hormones, to control the responses in other tissues. The two peptide hormone transcripts elevated under acute heat stress were POMC and PRL. POMC is predicted to have an appetite suppressive effect, which would reduce internal heat generation by metabolism. While the role of PRL in the chicken heat stress response is unclear, in mouse cells PRL reduces apoptosis and improves mitochondrial function in response to ER stress [68]. If these responses are similar in heat stressed chickens, PRL may reduce the negative effects of this stress.

As is typical in tissues responding to heat stress, there is enrichment of heat shock proteins and other heat responding genes that protect cells from misfolded and aggregated proteins. These transcripts encode chaperones and co-chaperones, such as members of the HSP and DNAJ gene families. Additional gene products that function as chaperones or co-chaperones include BAG3, CALR, CHORDC1, HERPUD1, POFUT2 and PLK3. In combination, the proteins encoded in these transcripts stabilize unfolded proteins, promote proper folding of nascent and unfolded proteins, inhibit aggregate formation, and mark proteins for proteasomal degradation.

Most chaperone and co-chaperone genes are expressed at a basal level in unstressed conditions forming a network maintaining protein homeostasis. Under heat stress, the transcription of these genes is increased to compensate for the challenge to protein folding caused by the elevated temperature. Several of the differentially expressed genes have additional functions beyond maintaining protein structural homeostasis. For example, PLK3 controls double stranded DNA break repair [69], regulates glucose metabolism [37], and is required for release from the G2 cell cycle checkpoint [70]. CALR plays a major role in cellular homeostasis by controlling calcium storage in the endoplasmic reticulum [25]. Furthermore, by interacting with chromatin, members of the HSPA gene family regulate cellular response to retinoic acid [32]. The multiple functions of these heat responsive genes allow the heat stress response to impact many different aspects of cell growth and metabolism.

A common response to heat stress is interruption of the cell cycle [71–74]. This is essential as heat stress induces oxidative stress which can cause DNA damage. Heat stress impacts the cell cycle depending upon which phase the cells were in when they began sensing heat. Typical responses include delaying exit from cell cycle checkpoints, particularly the G1/S or G2/M transitions and cells in S phase will prolong this phase prior to entering G2 [75–78]. Delaying exit from these phases allows DNA repair mechanisms to remove lesions caused by reactive oxygen species or other damaging agents. Of the transcripts upregulated by heat stress in the pituitary, HSPA2 [79] and KAT2A [80] regulate the G1/S checkpoint, HSPA5 [81] and GINS2 [82] regulate the G2/M checkpoint, and PLK3 controls the G1/S and G2/M checkpoints and can initiate cell cycle arrest [83].

Genes regulating both apoptosis and autophagy responded to heat stress in the pituitary. Overall, the majority of differentially expressed genes regulating these two processes favored promoting autophagy while suppressing apoptosis. Autophagy recycles cellular components and serves as a survival mechanism for cells under stress. In contrast, apoptosis is a cell death pathway that removes cells that have suffered significant damage. The elevated investment in autophagy versus apoptosis suggest that the pituitary cells of chickens subjected to 2 h of heat stress are in a survival mode.

In addition to driving improper protein folding, heat stress affects cellular membranes. One consequence of elevated temperature is an increase in membrane fluidity along with lipid raft reorganization. Lipid rafts are membrane microdomains that are enriched in cholesterol, sphingolipids, and saturated fatty acids. The distinct lipid composition of rafts allows these microdomains to interact with specific proteins. Consequently, changes in cholesterol composition of lipid rafts have been shown to affect the function of signaling proteins. For example, changes in the composition of the rafts can: alter the signaling capacity of GABA receptors [84], the ion gating function of nicotinic acetylcholine receptor [85], and agonist binding to serotonin receptors [86]. Heat stress significantly increased transcription of the SC5D gene that encodes the enzyme catalyzing the penultimate step in cholesterol synthesis. In addition, the expression of three other genes involved in cholesterol synthesis were elevated in the heat stressed pituitary. Further supporting the idea that heat stress increases cholesterol levels, DNAJA4 increases the stability and activity of HMG-CoA reductase protein, the rate-limiting enzyme in cholesterol synthesis. [20]. These observations imply that a consequence of pituitary heat stress is increased cholesterol production which likely affects lipid raft function. In neuronal cells members of the HSP90 and HSPA families, along with DNAJA4 localize to lipid rafts and reduction in cholesterol levels leads to loss of these proteins from the rafts [87]. Chaperones are responsible for maintaining signal transduction pathways in lipid rafts [88, 89], so changes in cholesterol level during heat stress likely modulates intercellular signaling.

Seven transcription factors were differentially regulated in the pituitary by heat stress. Their direct role in the heat stress response is uncertain, however several have functions that allow speculation. ID1, HR and ZBTB16 are proapoptotic transcription factors. ID1 and HR are down regulated by heat stress thus reducing their apoptotic activity. Elevated levels of ZBTB16 inhibit cell proliferation but promote apoptosis [90, 91]. ZBTB16 could inhibit cell proliferation during heat stress to permit the cell to recover through the actions of autophagy and DNA repair. In contrast, the proapoptotic ability of ZBTB16 could allow the protein to help clear the pituitary of severely damaged cells. ZMYND11 was also upregulated and the multidomain structure of the encoded protein allows it to regulate both transcription and splicing. Transcription is controlled by ZMYND11’s ability to bind Histone H3K36Me3. Once tethered to chromatin, ZMYND11 can either inhibit or promote the RNA polymerase elongation depending upon the targeted gene. With respect to splicing, ZMYND11 promotes intron retention through its interaction with U5 snRNP [66]. The biological function of retained introns is unclear although one speculation is that it coordinates gene expression [92, 93]. Finally, the upregulation of H2AFY2, which is typically associated with inactive chromatin, may help stabilize the down regulation of genes during heat stress.

In humans, a genome wide association study of gene expression identified genes expressing chaperones and heat shock proteins as having the highest heritability among the different ontology terms identified in the study [94]. Multiple studies in the chicken have shown significant heritability of various responses to heat stress including body temperature [95], feed efficiency, feed intake, [96], blood chemistry, [97] and meat yield [97]. Transcriptome studies such as the one reported here identify genes that are responsive to heat stress, some of which may be responsible for the heritable effects detected in genome wide association analyses. These will provide the target genes for either classical or modern genetic interventions to sustain livestock production traits in the face of climate change.

## Conclusion

The pituitary plays a central role in controlling the systemic response to environmental challenges such as heat stress. This work identified multiple pathways in the chicken’s pituitary affected by 2 h of heat stress including the chaperone, apoptosis, autophagy, cholesterol, lipid metabolic and cell cycle pathways. Transcripts encoding ubiquitinoylation, sumoylation, kinases, and histone acetylation enzymes were also differentially regulated as a function of heat stress. Included among the modifying enzymes were KAT2A and HR which encode histone acetylase and histone demethylase activity, respectively. Histone modifying enzymes have pleiotropic effects on gene expression patterns and likely play an important role in the heat stress response. Regulatory transcription factors and long non-coding RNAs were also modulated by thermal stress, which may contribute to subsequent heat stress responses. Finally, transcripts encoding the secreted hormones proopiomelanocortin and prolactin were both enriched by heat stress. These hormones have numerous systemic effects and likely are important to the organism’s overall response to thermal challenge.

The systemic response to heat stress is largely regulated by the hypothalamic–pituitary–adrenal axis. This study provides a partial view of the HPA axis with insight into the pituitary transcriptome, identifying the heat responsive genes in this tissue. A complete understanding of the HPA axis will need similar information from the hypothalamus and the adrenals. Ultimately, the goal is to provide a systems level description of the HPA’s response to thermal challenge. This information will provide understanding of how the systemic response to this stress is regulated, possibly suggesting interventions to improve poultry’s resilience to thermal challenge in the face of climate change.

## Supplementary Information

Below is the link to the electronic supplementary material.

Supplementary file1 (XLS 8691 KB)

## Data Availability

All transcriptome data sets are available through the GEO database: Accession: GSE89297.

## Abbreviations

ADO: Aminoethanethiol dioxygenase
AGPAT1: 1-Acylglycerol-3-phosphate O-acyltransferase 1
ANKRA2: Ankyrin repeat family A member 2
APOBEC2: Apolipoprotein B mRNA editing enzyme catalytic subunit 2
ASIC1: Acid sensing ion channel subunit 1
ASPN: Asporin
BAG3: BCL2 associated athanogene 3
BIVM: Basic, immunoglobulin-like variable motif containing
BLCAP: Bladder cancer associated protein
BLEC3: C-type lectin-like receptor 3
CALR: Calreticulin
CCDC126: Coiled-coil domain containing 126
CCNB3: Cyclin B3
CDK5R1: Cyclin dependent kinase 5 regulatory subunit 1
CFAP99: Cilia and flagella associated protein 99 cilia serve as sensory hubs
CHORDC1: Cysteine and histidine rich domain containing 1
COG4: Component of oligomeric golgi complex 4
CSPG5: Chondroitin sulfate proteoglycan 5
CSRNP1: Cysteine and serine rich nuclear protein 1
CSTA: Cystatin A
CUEDC1: CUE domain containing 1
CXCL14: C-X-C motif chemokine ligand 14
CYYR1: Cysteine and tyrosine rich 1
D26: Day 26 post-hatch
DEG: Differentially expressed genes
DNAJA1: DnaJ homolog subfamily A member 1-like
DNAJA4: DnaJ heat shock protein family (Hsp40) member A4
DNAJB1: DnaJ homolog subfamily B member 1-like
DNAJB5: DnaJ heat shock protein family (Hsp40) member B5
DPP10: Dipeptidyl peptidase like 10
EMC6: ER membrane protein complex subunit 6
FERMT2: Fermitin family member 2
FPKM: Fragments per kilobase per million (of reads mapped)
GABRA3: Gamma-aminobutyric acid type A receptor alpha3 subunit
GFPT2: Glutamine-fructose-6-phosphate transaminase 2
GFRA4: GDNF family receptor alpha 4
GINS2: GINS complex subunit 2
GNAT3: G protein subunit alpha transducin 3
GO: Gene Ontology
GSTAL1: Glutathione S-transferase alpha-like 1
GSX1: GS homeobox 1
HERPUD1: Homocysteine inducible ER protein with ubiquitin like domain 1
HR: Lysine demethylase and nuclear receptor corepressor
HS: Heat stress
HSD17B1: Hydroxysteroid 17-beta dehydrogenase 1
HSP: Heat Shock Protein
HSP90AA1: Heat shock protein 90 alpha family class A member 1
HSPA2: Heat shock 70 kDa protein 2
HSPA4L: Heat shock protein family A (Hsp70) member 4 like
HSPA5: Heat shock 70 kDa protein 5 (glucose-regulated protein
HSPA5: Heat shock 70 kDa protein 5 (glucose-regulated protein, 78 kDa)
HSPA8: Heat shock 70 kDa protein 8
HSPB8: Heat shock protein family B (small) member 8
HSPH1: Heat shock protein family H (Hsp110) member 1
ID1: Inhibitor of DNA binding 1
IFFO1: Intermediate filament family orphan 1
IFITM1: Interferon-induced transmembrane protein 1
IGLL1: Immunoglobulin lambda-like polypeptide 1
IL17REL: Interleukin 17 receptor E like
ITIH2: Inter-alpha-trypsin inhibitor heavy chain 2
JMJD6: Jumonji domain containing 6
KAT2A: Lysine acetyltransferase 2A
KBTBD4: Kelch repeat and BTB domain containing 4
LGALS8: Galectin 8
LNC: Long noncoding RNA
LOC101751708: Uncharacterized LOC101751708
LOC107049685: Uncharacterized LOC107049685
LOC107053492: Uncharacterized LOC107053492
LOC107054679: Uncharacterized LOC107054679
LOC112533635: Uncharacterized LOC112533635
LY86: Lymphocyte antigen 86
MACF1: Microtubule-actin crosslinking factor 1
MACROH2A2: MacroH2A.2 Histone
MAPKAP1: Mitogen-activated protein kinase associated protein 1
MC5R: Melanocortin 5 receptor
METTL27: Methyltransferase like 27
MTFP1: Mitochondrial fission process 1
MUC13: Mucin 13, cell surface associated
MUC22: Mucin-22-like LOC112530174
NANOS1: Nanos C2HC-type zinc finger 1
NKAIN3: Sodium/potassium-transporting ATPase subunit beta-1-interacting protein 3-like
NRN1: Neuritin 1
NRP2: Neuropilin 2
NSG1: Neuronal vesicle trafficking associated 1
OGN: Osteoglycin
P2RX2: Purinergic receptor P2X 2
P4HA2: Prolyl 4-hydroxylase subunit alpha 2
PCNA: Proliferating cell nuclear antigen
PHF6: PHD finger protein 6
PHLDA2: Pleckstrin homology like domain family A member 2
PIGC: Phosphatidylinositol glycan anchor biosynthesis class C
PITX2: Paired like homeodomain 2
PLK3: Polo like kinase 3
POFUT2: Protein O-fucosyltransferase 2
POMC: Proopiomelanocortin
PPP1R10: Serine/threonine-protein phosphatase 1 regulatory subunit 10-like
PRL: Prolactin
PRR5L: Proline rich 5 like
PRRG1: Proline rich and Gla domain 1
PSEUDOGENE_OTU1: PSEUDOGENE:OTU1
PTGES: Prostaglandin E synthase
Pseudo: SLC2A4RG:Pseudo: SLC2A4RG
RAB18L: Ras-related protein Rab-18-B-like
RALGAPA2: GTPase activating protein, alpha subunit 2 (catalytic)
RCBTB1: RCC1 and BTB domain containing protein 1
RNF128: Ring finger protein 128, E3 ubiquitin protein ligase
SC5D: Sterol-C5-desaturase
SCAMP3: Secretory carrier membrane protein 3
SENP1: SUMO specific peptidase 1
SGCG: Sarcoglycan gamma
SKP2: S-phase kinase associated protein 2
SLC16A9: Solute carrier family 16 member 9
SMAD2Z: SMAD family member 2-Z
SMPD3: Sphingomyelin phosphodiesterase 3
SOSTDC1: Sclerostin domain containing 1 (BMP) antagonist
SOX17: SRY-box 17
STAG1: Stromal antigen 1
SULT2B1L1: Sulfotransferase family cytosolic 2B member 1-like 1
SZRD1: SUZ RNA binding domain containing 1
TBX18: T-box 18
TBX22: T-box 22
TECR: Probable very-long-chain enoyl-CoA reductase art-1
TMEM159: Transmembrane protein 159
TMEM259: Transmembrane protein 259
TN: Thermoneutral
TP53INP2: Tumor protein p53 inducible nuclear protein 2
TPM4: Tropomyosin 4
TUBD1: Tubulin delta 1
USP25: Ubiquitin specific peptidase 25
USP9X: Ubiquitin Specific Peptidase 9 X-Linke
VIPAS39: VPS33B interacting protein
WWP1: WW domain containing E3 ubiquitin protein ligase 1
ZBTB16: Zinc finger and BTB domain containing 16
ZBTB46: Zinc finger and BTB domain containing 46
ZDHHC2: Zinc finger DHHC-type containing 2
ZMYND11: Zinc finger MYND-type containing 11
ZMYND11: Zinc finger MYND-type containing 11
ZNF207: Zinc finger protein 207
qRT-PCR: Quantitative reverse transcription polymerase chain reaction
